# Implementation of GeneXpert for TB Testing in Low- and Middle-Income Countries: A Systematic Review

**DOI:** 10.9745/GHSP-D-21-00121

**Published:** 2021-09-30

**Authors:** Scott Brown, Justine E. Leavy, Jonine Jancey

**Affiliations:** a School of Public Health, Curtin University, Perth, Western Australia, Australia.

## Abstract

**Introduction:**

Current evidence indicates that the impact of GeneXpert for diagnosing TB in low- and middle-income countries (LMICs) has not demonstrated equivalent outcomes when compared to Xpert evaluations in upper-middle-income countries. Challenges associated with implementation are possible contributing factors preventing this innovative diagnostic technology from achieving more significant public health outcomes. This review aimed to assess the use of implementation science frameworks when reporting the enablers and barriers for the implementation of GeneXpert for diagnosing TB in LMICs.

**Methods:**

We conducted a qualitative systematic review of the peer-reviewed literature using PubMed, Medline, and Scopus. Eligible articles were those published between January 2010 and March 2020 that identified enablers and barriers to GeneXpert implementation, as well as the implementation approach delivered in an LMIC.

**Results:**

Eleven studies were included in the review. Implementation barriers were found to be relatively consistent across studies and included patient-level factors, human resources, material resources, service implementation, service coordination, and technical operations. Few studies (n=5) identified enabling factors in the implementation of Xpert for TB testing. Identified enablers included strategies such as active case finding, expanding diagnostic algorithms, and daily transport of samples. The public health impact of Xpert TB testing interventions was commonly influenced by implementation barriers (n=4). Of the 11 studies, only 3 reported against an implementation framework.

**Conclusion:**

This review identified a commonality in implementation barriers and enablers that influenced the overall public health impact of GeneXpert. With greater transparency of these barriers and enablers, program planners can promote a more collaborative approach and adapt interventions. It is recommended that program planners use implementation science frameworks when conducting research and publishing. This will build an evidence base focused on implementation and thereby support programs to address implementation barriers and include enabling factors in program design.

## Abstract (precis)

This review highlights a commonality of implementation barriers across geographically dispersed GeneXpert interventions for TB testing. This indicates the importance of using implementation frameworks to report findings that can improve public health outcomes across low- and middle-income countries.

## Key Messages

- In low- and middle-income countries (LMICs) that have a higher burden of TB, the necessity for improved and rapid diagnostic testing, such as Xpert MTB/RIF that detects both TB and rifampicin resistance, has been limited by resource-related implementation challenges.
- Identifying and responding to the implementation-related barriers and enablers in Xpert testing programs across LMICs can help improve public health outcomes, which in this review were negatively impacted by barriers that were identified.
- We found that across the studies included in the review, an integrated and coordinated approach was required for implementing Xpert TB models of care into different health settings, but in LMICs, this approach can be associated with a lack of integration and coordination.
- When deciding on a particular implementation approach, the existing context of a health setting and the expertise and needs of key stakeholders (for example, clinicians, laboratories, and government) should be considered.
- Reporting of Xpert programs against an implementation science framework can increase learning across geographically diverse settings and thereby support improvement in program design.

## INTRODUCTION

An estimated 10 million people were affected by TB globally in 2019, with the total number of deaths reaching 1.2 million people,^1^ down from 1.5 million people in 2018.^2^ Although TB is prevalent in all countries, the distribution shows a significant burden in low- and middle-income countries (LMICs). It is estimated that up to two-thirds of global incident cases are found in only 8 countries, 5 of which are classified as LMICs.^1^ In 2019, the highest proportion of new cases (44%) occurred in Southeast Asia.^1^^,^^3^

In addition, approximately half a million cases of rifampicin-resistant TB were diagnosed in 2019.^1^^,^^4^ Of these cases, 78% had multidrug-resistant TB (MDR-TB).^1^ It has also been estimated that up to one-third of global TB cases and more than three-quarters of MDR-TB cases are undetected,^3^ equating to upward of 3.3 million people globally living with active TB who are unaware of their status and remain undiagnosed.^5^

The global public health response to TB is guided by the World Health Organization (WHO) *End TB Strategy*^2^^,^^6^ and the Sustainable Development Goals.^2^ Both of these strategic documents prioritize TB as a health issue, aiming to achieve a 95% reduction in TB deaths and 90% reduction in the incidence rate by 2035.^6^ Achieving these goals will require a high level of collaboration between regional, national, and international stakeholders working in partnership across a range of interventions targeting TB risk factors and priority populations.^7^ These often include interventions that aim to reduce the time to diagnosis, provide more effective contact tracing, improve treatment adherence and outcome, enhance collaborative partnerships with HIV-specific programs, and prevent TB transmission.^3^

Case detection interventions are an ongoing challenge,^5^ often due to restrictive testing algorithms that reduce access to testing among people with presumptive TB who fall outside the scope of the algorithm. However, in resource-limited settings, removing these restrictions comes with significant cost implications.^8^ Therefore, no single intervention will result in achieving the global TB elimination targets.^7^ Modelling undertaken to predict the impact of a variety of interventions indicates that improvements in diagnostic testing will make a substantial contribution.^7^ Earlier detection of TB and MDR-TB allows cases to be promptly triaged into appropriate treatment and care, leading to improved patient outcomes.^3^^,^^9^ When comparing point-of-care testing approaches with more conventional laboratory-based testing procedures, the longer timeframe for delivery of laboratory results is often cited as a reason for disengagement from the treatment pathway,^10^ with recent estimates suggesting only 56% of diagnosed MDR-TB cases worldwide are treated successfully.^2^

Case detection interventions are an ongoing challenge, often due to restrictive testing algorithms that reduce access to testing among people with presumptive TB who fall outside the algorithm’s scope.

In 2010, the WHO recommended the use of the Xpert MTB/RIF (Cepheid, Inc., Sunnyvale, CA), a test that simultaneously detects Mycobacterium tuberculosis (MTB) and rifampicin-resistant TB (RIF) strains, as the initial diagnostic for people presumed to have MDR-TB or HIV-associated TB in high-incidence countries.^3^^,^^11^ These recommendations have since expanded to all people with presumed TB while acknowledging resource implications within resource-limited settings.^12^ The Xpert advances a health system's ability to diagnose and respond to TB as it has improved sensitivity in comparison to sputum smear microscopy.^5^ Studies indicate that Xpert can detect TB in a large number of people that routine testing services cannot detect.^13^ Therefore, new models of care are possible due to this rapid diagnostic technology that returns results within 2 hours.

Since the recommendation of Xpert, the number of programs using this new diagnostic technology to improve access to testing services has increased significantly.^14^ By 2017, 23 million testing cartridges had been procured for use across 6,659 Xpert machines located in 130 countries.^11^^,^^15^ However, requirements for specialist staff, temperature control, and a continuous power supply, as well as the high cost of purchasing the machine and ongoing testing cartridges, restrict the installation of Xpert in many locations.^11^

The challenges associated with implementation differ significantly between high-income and low-income countries.^5^^,^^16^ The impact of new diagnostic testing interventions is often dependent on the functioning of the overall system in which the intervention is being introduced, as well as the operational implementation of the Xpert program.^7^^,^^17^^,^^18^ In LMICs, implementation challenges often impact the effectiveness of Xpert, with many failing to achieve the expected outcomes demonstrated by Xpert evaluations in upper-middle-income countries.^5^ This includes indicators such as a reduced timeframe to deliver test results to patients or successfully transitioning newly diagnosed cases into quality treatment and care.^14^^,^^17^^,^^19^ As countries begin to scale up Xpert-based interventions, understanding how contextual factors influence program impact is critical.^20^

As countries begin to scale up Xpert-based interventions, understanding how contextual factors influence program impact is critical.

*Implementation research is the scientific study of methods to promote the systematic uptake of research findings and other evidence-based practices into routine practice, and hence, to improve the quality and effectiveness of health services and care.* ^21^

Furthermore, it explores the impact that implementation has on outcomes and identifies sustainable improvements.^22^ This approach recognizes that challenges often experienced in real-world health settings impede meaningful outcomes for interventions that were previously proven effective in research studies.^23^ For this reason, sharing knowledge from across Xpert sites is needed to improve real-world implementation and program outcomes.^13^ With a higher burden of TB in LMICs, implementation research in this setting can advance understanding of how the challenges of implementation impact effectiveness.

With a higher burden of TB in LMICs, implementation research in this setting can advance understanding of how the challenges of implementation impact effectiveness.

This review aimed to assess the use of implementation science frameworks when reporting the enablers and barriers for the implementation of Xpert for the diagnosis of TB in LMICs. The specific objectives were to (1) identify approaches to implementing Xpert in LMICs, (2) determine the barriers and enablers across the identified Xpert testing programs, and (3) assess the use of implementation science frameworks in Xpert programs in LMICs.

## METHODS

This qualitative systematic review was developed in accordance with the Preferred Reporting Items for Systematic Reviews and Meta-Analyses (PRISMA) statement, which ensures transparency in the formulation of findings.^24^ The data analysis consisted of de-identified, publicly available data and therefore is exempt from ethics approval.

### Eligibility Criteria

The PICOS (population, interventions, comparisons, outcomes, and study design) was used to frame the research question. The population was limited to LMICs as defined by the World Bank classification of low-income economies or lower-middle-income economies.^25^ This was in recognition of the unique challenges associated with implementing Xpert within this setting.^5^^,^^16^ Within this population setting, interventions that used the Xpert technology for TB testing in public or private sector facilities were included. In the data analysis process, a comparison of implementation approaches used across the included studies was undertaken. Therefore, a within-study comparison group was not required to meet eligibility. The outcome variables of interest were the identified enablers and barriers to implementation, as well as the implementation approach.

As the Xpert was recommended for use by the WHO in 2010, articles were limited to those published from January 1, 2011, to March 31, 2020. Included studies were peer-reviewed and published in English where the full-text article was available. Articles were excluded if they were not single country reviews and if they did not specify the implementation approach as well as identifying barriers and/or enablers to the implementation approach. Further to this, articles focused on the implementation of Xpert for TB in closed population settings, such as prisons or pediatric clinics, multicountry reviews, and interventions occurring under a research trial were also excluded.

### Search Strategy and Study Selection

To identify relevant literature, PubMed, Medline, and Scopus databases were searched over 3 weeks beginning April 4, 2020. Search terms included the keywords “implementation,” “GeneXpert OR Xpert” and “Tuberculosis OR TB.” The preliminary search was performed in PubMed.

In total 2,296 articles were initially identified. Articles were extracted to EndNote X9.3.2 and 116 duplicates were removed. All articles identified underwent a review to assess relevance against the eligibility criteria. This review process occurred in stages, which initially involved screening the article titles (excluding 2,029 records). Then, the abstracts of the remaining 151 articles were assessed to identify those suitable for full-text review. In completing this process, a further 133 articles were excluded. This resulted in 18 articles undergoing an eligibility assessment involving a full-text review. A total of 7 articles were excluded as they did not address the barriers and/or enablers in the implementation of the intervention. A second full-text review process was then completed involving all 3 authors, who assessed the remaining articles against the eligibility criteria and mutually agreed on the final selection of studies for the data extraction process. A total of 11 articles were included in the review (Figure).

**FIGURE:**
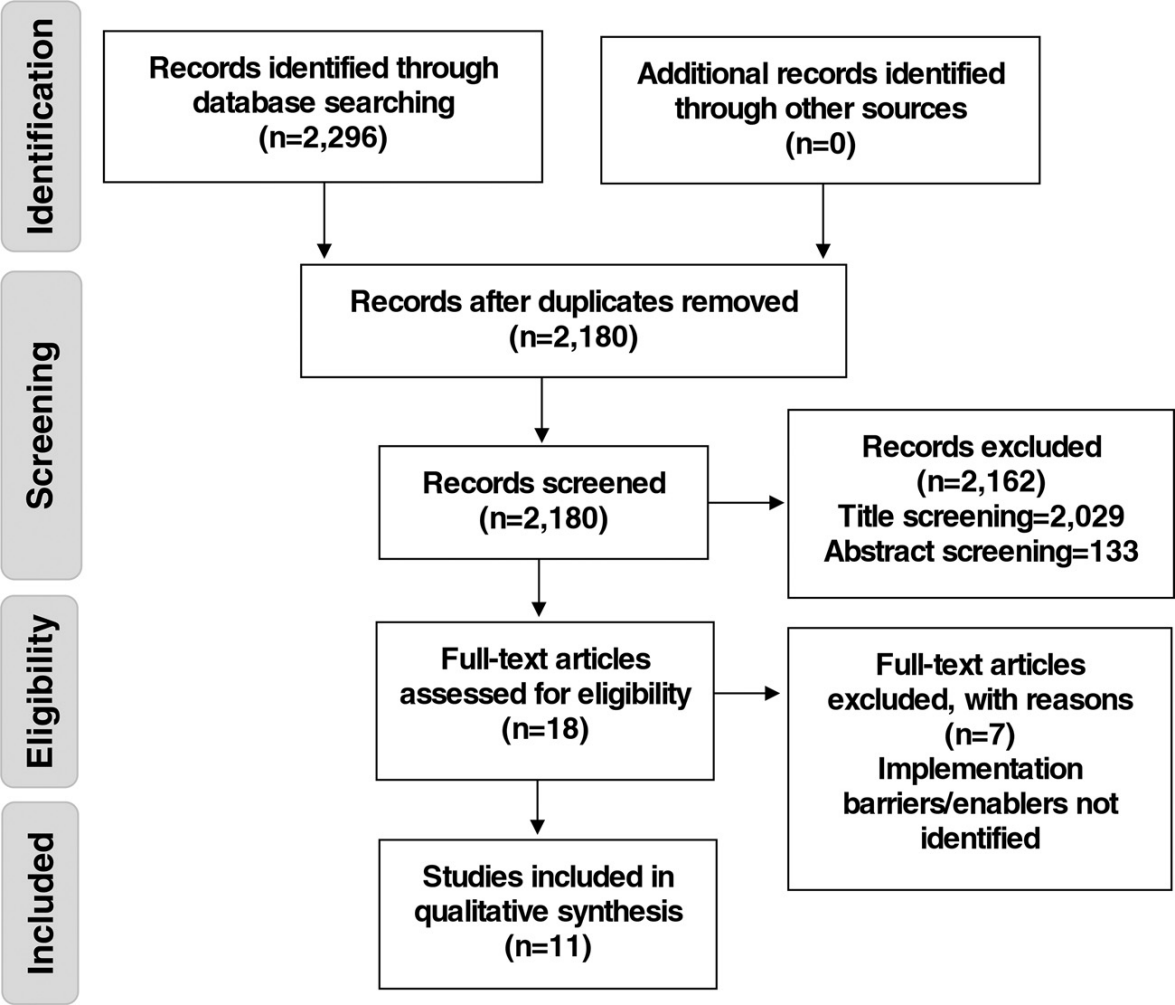
PRISMA Flow Chart for Study Selection on Implementation of GeneXpert for TB Testing in Low- and Middle-Income Countries Abbreviation: PRISMA, Preferred Reporting Items for Systematic reviews and Meta-Analyses.

### Data Extraction and Analysis

Data were independently extracted from selected articles and transferred into an Excel spreadsheet to maintain consistency. Before the data extraction process, variables for extraction were agreed upon among all authors. The final variables included author, year, country of implementation, implementation approach (i.e., the characteristics of how an intervention is implemented, for example, a hub-and-spoke model or a point-of-care model), health setting, testing algorithm, public health impact, as well as the identified barriers and enablers to implementation and use of an implementation framework. The hub-and spoke-model was characterized by a centrally located laboratory performing the TB testing using the Xpert machine and returning the results to the community health clinic that initially collected the sputum sample. The point-of-care model involved the installation of the Xpert machine at health centers where sputum samples were collected and tested.

## RESULTS

### Study Characteristics

The final 11 articles were published between 2015 and 2020, with Xpert implementation occurring in Asia (n=5) and Africa (n=6). More details on the characteristics of the 11 studies are summarized in Table 1.

**TABLE 1.** Characteristics of Studies on Implementation of Xpert for TB Testing in Low- and Middle-Income Countries

**Author (Year)**	**Country**	**Implementation Approach**	**Health Setting**	**Testing Algorithm**	**Public Health Impact**
Cattamanchi et al.^15^ (2020)	Uganda	Hub-and-spoke model: Testing ‘hubs’ linked to 3–5 microscopy unit “spokes” Monitoring of results centralized through National TB Reference Laboratory.	Regional or district public hospital for testing, with community health center for sample collection	Any person with presumed TB.	Nearly 4-fold increase in confirmed MDR-TB from 2009–2017 Increase in TB CNR from approximately 41,000 cases pre-2010 to 57,756 cases in 2017
Cowan et al.^26^ (2015)	Mozambique	Hub-and-spoke model:Xpert installed in 4 public hospitals in 4 districts. Transportation network established from select health centers to transfer samples for testing.	District and urban public hospital for testing. Urban and remote health centers for sample collection	Two-step algorithm for people suspected of having pulmonary TB. GeneXpert testing occurs after 2 separate smear-negative results using smear microscopy.	Increase in diagnosis of bacteriologically confirmed pulmonary TB by 69%
Sikhondze et al.^27^ (2015)	Swaziland	Hub-and-spoke model:23 Xperts installed in 19 TB diagnostic laboratories. Community health sample transportation covers 78% of country. NGO covers the remaining regions.	TB diagnostic laboratories for testing	Not stated.	Not stated.
Nalugwa et al.^28^ (2020)	Uganda	Hub-and-spoke model.249 Xpert machines in 227 of 1500 TB diagnostic units. Motorcycle riders employed by Central Public Health Laboratories transport samples from community health centers.	Regional or district public hospital testing hub, with community health center for sample collection	At time of study, Xpert testing available to PLHIV, health care workers, contacts of DR-TB, pregnant women or breastfeeding mothers, prisoners, patients from refugee camps, and diabetics.	Not stated.
Newtonraj et al.^29^ (2019)	India	Hub-and-spoke model:Xpert installed at the Intermediate Reference Laboratory in a government hospital for chest diseases in Puducherry district. Samples are received from 27 designated microscopy centers in medical colleges or district-level hospitals.	Centralized testing in Intermediate Reference Laboratory within government hospital for chest diseases. Sample collection from district hospitals.	Initial diagnostic for extrapulmonary, pediatric, and HIV-associated TB. Xpert is also an add-on test for sputum microscopy negative patients.	CNR reduced from 118 to 97 per 100,000 population between 2010 and 2017
Rendell et al.^30^ (2017)	Mongolia	Hub-and-spoke model:3 Xpert machines installed across the country. Samples collected at community TB clinics and results returned using several paper-based delivery options.	Testing available at the National TB Reference Laboratory, the Regional Diagnostic and Treatment Centre, and a northern province hospital. Samples collected at community/district level TB clinics.	All smear-negative pulmonary TB cases, patients with presumed TB diagnosed with HIV, patients with presumed MDR-TB, and all smear-negative new cases aged 15–35 years	Number of diagnosed cases increased from 2,783 in 2012 to 3,209 in 2015
Gidado et al.^31^ (2018)	Nigeria	Point-of-care model: 176 Xperts installed at clinics that meet necessary installation requirements. Test results monitored centrally, as well as the procurement of supplies.	Primary, secondary, and tertiary facilities	Not stated.	Not stated.
Hoang et al.^32^ (2015)	Vietnam	Point-of-care model: Xpert installed in TB units of district health center in 35/63 provinces. Provinces chosen based on known prevalence of MDR-TB and/or HIV.	TB units in district health centers	Presumptive MDR-TB cases, defined as belonging to a risk category including TB treatment non-converters; contact of a person with MDR-TB; person coinfected with TB/HIV; >1 month using TB drugs.	37.8% of estimated presumptive MDR-TB patients tested 75% of identified MDR-TB patients completed treatment and cured.
Joshi et al.^33^ (2018)	Nepal	Point-of-care model: Xpert installed in 26 health facilities under TB Reach Project and operated by either government or NGO. In 2014, all machines donated to government. Samples collected from patients for smear microscopy, and an additional sample collected for Xpert testing, where available.	Government health facilities such as District Public Health Office laboratory, hospital and primary health centers located throughout the country	Targeted to specific populations as per WHO recommendations, including children aged younger than 15 years, PLHIV, severe forms of TB, and in presumptive MDR-TB.	Xpert diagnosed 28% of the total bacteriologically confirmed TB cases in 2015/2016.
Mustapha et al.^34^ (2015)	Nigeria	Point-of-care model: Xpert implemented at 22 sites by NGO in partnership with government. Governance oversight by the National TB Control Program in the form of an advisory committee.	10 secondary health facilities, 10 tertiary hospitals, 2 private health facilities	Targeted to specific risk groups, including PLHIV with presumptive TB, those with poor response/relapse to TB treatment, contact of known MDR-TB case, TB cases at risk of resistance.	Not stated.
Awan et al.^35^ (2018)	Pakistan	Two-pronged point-of-care approach: A “private-public mix model” with an Xpert installed at the TB lab of 6 public hospitals and 1 private site participating in Programmatic Management of Drug-Resistant TB. Active case finding occurred among outpatients and in wards of hospitals. The second “social business model” introduced Xpert at 3 TB centers for testing, with community screeners identifying symptomatic patients from nearby private-sector clinics and referring them to TB clinics.	Public and private hospitals and private community clinics	Initial diagnostic for people with presumptive TB.	43% increase in diagnosed DR-TB 83.2% of TB cases found in the public-private mix model.

Abbreviations: CNR, case notification rate; DR-TB, drug-resistant TB; MDR-TB, multi-drug resistant TB; NGO, nongovernmental organization; PLHIV, people living with HIV.

### Implementation Approaches

Among the studies included in this review, the hub-and-spoke implementation approach for Xpert was used more often (n=6)^15^^,^^26^^–^^30^ than the point-of-care model (n=5).^31^^–^^35^ As part of the hub-and-spoke model, varying approaches to the transportation of samples between the laboratory and clinic were identified, which included transportation by a health care worker,^30^ employed drivers,^26^^,^^28^ and informal couriers.^27^ The return of results to the clinic that collected the patient sputum sample most commonly occurred via the same transport network, while a study in Uganda examined the use of SMS to return results directly to the clinic and patient.^15^ Of the remaining interventions implementing the point-of-care model, one had 2 distinct arms involving differing approaches in active case finding of symptomatic people.^35^ This included health workers actively seeking sputum samples from hospital patients or community-based screeners referring symptomatic people attending private health clinics to a TB testing center with an Xpert machine installed.

### Health Setting

For the hub-and-spoke models (n=6), the Xpert machine was installed at a combination of high level facilities such as regional (n=2), district (n=3) and urban (n=1) public hospitals (n=4),^15^^,^^26^^,^^28^^,^^29^ or dedicated national TB diagnostic laboratories (n=2).^27^^,^^30^ Information relating to the possible co-location of TB laboratories within hospitals was not provided. The majority of the spokes in these studies were community-level primary health clinics (n=4)^15^^,^^26^^,^^28^ or a TB community clinic (n=1).^30^ For the point-of-care implementation models, the Xpert was installed at health settings defined as primary health care sites (n=2),^31^^,^^33^ secondary-level facilities (n=2),^31^^,^^34^ dedicated TB units/public health office (n=3)^27^^,^^32^^,^^33^ and hospitals (n=4).^31^^,^^33^^–^^35^ Two studies located an Xpert in a private health facility.^34^^,^^35^

### Testing Algorithm

The testing algorithm that determined eligibility for an Xpert test varied across all interventions. Although the majority used Xpert as an initial diagnostic for their targeted population (n=6),^15^^,^^28^^,^^29^^,^^33^^–^^35^ 3 studies implemented Xpert as a secondary diagnostic after smear-negative microscopy or a combination of both.^26^^,^^29^^,^^30^ Across all studies, the broadest testing algorithm was defined as any person with presumptive TB (n=2).^15^^,^^35^ The remaining studies provided Xpert testing to a combination of people deemed to be within an at-risk category, such as people living with HIV,^28^^,^^33^^,^^34^ children aged 15 years or younger,^33^ people with presumptive MDR-TB,^32^^,^^33^ a contact of a known DR/MDR-TB case,^28^^,^^32^^,^^34^ TB cases at risk of resistance,^32^^,^^34^ health workers,^28^ pregnant women or breastfeeding mothers,^28^ prisoners,^28^ refugees,^28^ and diabetics.^28^ One study limited the testing algorithm to cases of presumptive MDR-TB, as the intervention was focused on identifying MDR-TB cases.^32^ Two studies did not indicate the testing algorithm used, both of which primarily focused on the technical use of the Xpert machine and identifying the cause of error in results.^27^^,^^31^

### Public Health Impact

Seven of the 11 studies outlined some form of public health impact achieved by the implementation of Xpert.^15^^,^^26^^,^^29^^,^^30^^,^^32^^,^^33^^,^^35^ Three studies assessed the Xpert within a continuum of care and highlighted treatment-related public health impacts within the cohort of TB cases detected by Xpert.^15^^,^^26^^,^^32^ For example, the primary outcome identified by Hoang et al.^32^ was the successful treatment of 75% of the identified MDR-TB cases, having tested 31.2% of the estimated MDR-TB cases nationally. This study, along with the Nepalese study,^33^ framed the Xpert specific implementation impact as the proportion of national cases identified by Xpert. A limited number of studies (n=5) identified a measurable public health impact relating directly to TB testing using Xpert.^15^^,^^26^^,^^29^^,^^30^^,^^35^ These included an increase in the identified cases of TB (n=3),^15^^,^^30^^,^^35^ DR-TB (n=1)^35^ or MDR-TB (n=1),^32^ and 1 study saw a decline in the TB case notification rate from 118 to 97 per 100,000 people between 2010–2017^29^ (see Table 1).

### Implementation Barriers

The barriers were allocated into the following 6 categories: patient-level factors, human resources, material resources, service implementation, service coordination, and technical operations (Table 2). Of the total (n=28) barriers identified, 43% (n=13) were found to have occurred in multiple studies.

**TABLE 2.** Identified Barriers to Implementing Xpert TB Testing in Studies in Low- and Middle-Income Countries

**Barriers**	**India**	**Mongolia**	**Mozambique**	**Nepal**	**Nigeria**	**Pakistan**	**Swaziland**	**Uganda**	**Vietnam**
Patient-level factors									
Distance to testing sites^29^^,^^33^^,^^35^	X			X		X			
Cost of testing in private health clinics^35^						X			
Human resources									
Inadequate/inconsistent staff training on testing processes/guidelines, and/or limited awareness of availability^26^^,^^28^^–^^30^^,^^32^^,^^34^^,^^35^	X	X	X		X	X		X	X
Low self-efficacy and confidence that Xpert improves outcomes^15^								X	
Workload capacity in laboratories^15^^,^^30^		X						X	
High staff turnover^15^^,^^31^^,^^32^					X			X	X
Initial struggle with English software (since rectified)^26^			X						
Material resources									
Inadequate power supply^15^^,^^26^^,^^27^^,^^31^^,^^33^^,^^35^			X	X	X	X	X	X	
Poorly equipped labs (e.g., limited space for patient assessment; no ventilation, workbench, air conditioning, and/or refrigerator)^15^^,^^26^^,^^34^			X		X			X	
Inappropriate storage of cartridges^31^					X				
Service implementation									
Geographically dispersed TB laboratories^35^						X			
Transportation of sputum samples (e.g., inconsistent/delays in availability of deliveries; improper packaging/temperature control during transport)^15^^,^^32^^,^^34^^,^^35^					X	X		X	X
Inability to track/follow up with patients testing positive^15^								X	
Determining an appropriate testing algorithm^26^			X						
Failure to identify eligible cases for screening^30^^,^^32^		X							X
Limitations to accessing updated and clear standard operating procedures/internal audits^27^^,^^32^							X		X
Poor quality samples collected^27^^,^^29^^,^^30^^,^^32^	X	X					X		X
Service coordination									
Supply chain for procurement of cartridges, reagents, and/or medicines resulting in lack of supplies^15^^,^^26^^,^^30^^,^^32^^–^^35^		X	X	X	X	X		X	X
Lack of referral pathways/communication between staff and health centers (e.g., referral pathways and transfer of results)^27^^–^^30^^,^^32^^–^^35^	X	X		X	X	X	X	X	X
Insufficient oversight from national body/remote monitoring^15^^,^^26^			X						X
Delays in notification of results^15^^,^^26^^,^^28^^–^^30^^,^^34^	X	X	X		X			X	
Limited ability to track positive cases and confirm treatment^26^^,^^28^^,^^35^			X			X		X	
Technical operations									
Xpert maintenance (e.g., frequency of maintenance not always implemented as required; poor understanding of routine maintenance in dusty, non-temperature-controlled labs)^26^^–^^28^^,^^31^^,^^33^^,^^35^			X	X	X	X	X	X	
Failure of calibration and required replacement^26^			X						
Lack of timely replacement of damaged modules^33^				X					
Module malfunction^28^^,^^31^					X			X	
Limited internet connectivity^26^^,^^31^			X		X				
Local repair options limited^30^		X							

The greatest number of barriers were categorized as service implementation factors (n=7). However, the most cited barrier was a service coordination factor (n=8) involving the lack of communication/referral pathways between staff in laboratories and health centers.^27^^–^^30^^,^^32^^–^^35^ The second most common barrier related to inadequate and/or inconsistent staff training, which was identified relatively equally by studies in both Africa (n=3)^26^^,^^28^^,^^34^ and Asia (n=4).^29^^,^^30^^,^^32^^,^^35^

The greatest number of barriers were categorized as service implementation factors; however, the most cited barrier was a service coordination factor.

Overall, the hub-and-spoke model generated the most commonly occurring barriers. Of the studies that implemented this approach, Cowan et al.^26^ was the only study that did not identify communication barriers between the testing laboratory and the clinic collecting patient samples. Further to this, delays in patient notification of results were also a common barrier associated with the hub-and-spoke model (n=5).^15^^,^^26^^,^^28^^–^^30^

### Implementation Enablers

Five studies^15^^,^^30^^,^^32^^,^^33^^,^^35^ identified enabling factors in the implementation of Xpert for TB testing, including strategies such as taking an active case-finding approach,^35^ expanding diagnostic algorithms,^35^ and the daily transport of samples^15^ (Table 3). Of the studies that identified enablers, only 2 highlighted more than 2 factors.^30^^,^^35^ Of the enablers identified, the single factor highlighted by more than 1 study was the addition of human resources to support the implementation of a new program.^30^^,^^35^ For the study conducted in Mongolia, this corresponds with the identified barrier that relates to the workload capacity of laboratory staff.^30^ Further enablers have a correlation with an identified barrier; for example, Uganda stated the daily transport of samples as an enabling factor, while also identifying inconsistent and/or delayed transportation of samples as a barrier to successful implementation.^15^

**TABLE 3.** Identified Enablers of Implementation of Xpert TB Testing Studies in Low- and Middle-Income Countries

**Enablers**	**Country**
Daily transport of samples^15^	Uganda
SMS communication of results to health centers^15^	Uganda
Collecting monthly performance feedback from staff for quality improvement purposes^15^	Uganda
Clear guidelines in local language^30^	Mongolia
Purchase of uninterruptable power supply^30^	Mongolia
Access to external experts^30^	Mongolia
Peer learning for professional development^30^	Mongolia
Consistent process of confirming of results between referring site and laboratory after sample sent^32^	Vietnam
Laboratory personnel understood Xpert to be superior to smear microscopy^33^	Nepal
Active case finding approach^35^	Pakistan
Expanded diagnostic algorithm^35^	Pakistan
Additional human resources^30^^,^^35^	Pakistan, Mongolia
Close collaboration^35^	Pakistan
Supervisory visits to improve maintenance and stock procurement^35^	Pakistan

Abbreviation: SMS, short message service.

Of the enablers identified, the single factor highlighted by more than 1 study was the addition of human resources to support the implementation of a new program.

### Implementation Science

Of the studies included in this review, only 3 reported being guided by a structured reporting framework,^27^^,^^30^^,^^33^ and only 1 was specifically for implementation studies.^33^ These included the Strengthening the Reporting of Observational studies in Epidemiology (STROBE),^27^ the Consolidated Criteria for Reporting Qualitative Research,^30^ and the Standards for Reporting Implementation Studies (StaRI).^33^

## DISCUSSION

This review aimed to assess the use of implementation science frameworks when reporting approaches to implementing Xpert in LMICs and determining the barriers and enablers across the identified Xpert testing programs. The review found 7 of the 11 identified studies outlined some form of public health impact achieved by Xpert, which included treatment success (n=3)^15^^,^^26^^,^^32^ and an increase in identification of active cases (n=3).^15^^,^^30^^,^^35^ Mostly consistent barriers to implementing Xpert were reported across all the identified studies. This highlights a commonality of implementation barriers across geographically dispersed Xpert interventions in LMICs. In contrast, less than half of the studies articulated enabling factors in the implementation of Xpert for TB testing in LMICs. More consistent and transparent reporting using implementation science frameworks will improve access to information that supports improved public health outcomes for real-world Xpert interventions in LMICs.^13^

### Implementation Approach

In this review, we found an integrated and coordinated approach was required when implementing Xpert models of care into a health setting.^5^^,^^28^ This was particularly apparent in the implementation of the commonly used hub-and-spoke model. In high-income countries, this model of care is considered ideal for maximizing efficiencies and effectiveness for services that require advanced medical equipment, such as the Xpert.^36^ However, in LMICs the hub-and-spoke model was found to be associated with the greatest number of barriers, highlighting a lack of integration and service coordination. Therefore, when deciding on a particular implementation approach, the existing context of a health setting and the expertise and needs of key stakeholders (e.g., clinicians, laboratories, and government) should be considered.^37^

Recommendations to enhance integration and coordination included supporting continuous quality improvement of systems, as well as procuring and maintaining appropriate equipment, strengthening supply chains, having reliable specimen referral networks, suitable laboratory information systems, and proper laboratory training for staff.^37^ This is reinforced by Rendell et al.,^30^ indicating that the mere introduction of Xpert does not automatically “guarantee a natural fit into the [existing] program environment.”^30^ There is a need for more consistent, transparent, and collaborative information sharing regarding the suitability of strategies to support the implementation and integration of Xpert programs at the local level.^37^

### Barriers to Greater Public Health Impact

Across all the identified studies, 2 main barriers to the identification of active TB cases were identified, the underutilization of Xpert and the inadequate identification of eligible patients. These barriers frequently resulted from a lack of communication/referral pathways between health centers and laboratories (n=8)^27^^–^^30^^,^^32^^–^^35^ and inadequate or inconsistent training to support staff awareness and knowledge of testing and/or testing processes (n=7).^26^^,^^28^^–^^30^^,^^32^^,^^34^^,^^35^ Restricted testing algorithms were also likely to have affected the identification of eligible patients, as this adds a level of complexity in triage that requires specific staff training, which was lacking; therefore, staff were not adequately identifying eligible patients. Furthermore, the underutilization of Xpert was exacerbated when stocks of material resources such as sputum cups for sample collection were not maintained; 7 studies had periods of downtime due to inadequate supplies.^15^^,^^26^^,^^30^^,^^32^^–^^35^ These barriers are also reflected in multicountry reviews that analyzed quarterly reports and machine data.^13^

Two main barriers to the identification of active TB cases were identified, the underutilization of Xpert and the inadequate identification of eligible patients.

As the Xpert allows for the timely turnaround of test results, a secondary public health impact of Xpert is the immediate initiation of treatment to prevent onward transmission.^33^ However, in this review, 8 studies identified a delay in either samples being delivered for testing (n=4)^15^^,^^32^^,^^34^^,^^35^ or a delay in the notification of test results (n=6).^15^^,^^26^^,^^28^^–^^30^^,^^34^ Accordingly, a delay in the notification of results reduces the opportunity to improve treatment initiation.^16^^,^^26^ In examining the difference in time to treatment between patients diagnosed by Xpert and those diagnosed under the previous smear microscopy system, several studies (n=2) found that an Xpert diagnosis resulted in a longer time to treatment or loss to follow-up.^13^^,^^26^^,^^28^ These outcomes are often noted as the result of operational challenges associated with program implementation.^17^ With consistent reporting of barriers that inhibit the integration of Xpert into the existing health system, the overall public health impact of Xpert implementation in LMICs can be improved.^16^

### Implementation Enablers

In this review, only 5 studies specifically identified enablers for the implementation of Xpert for TB testing in LMICs.^15^^,^^30^^,^^32^^,^^33^^,^^35^ Identifying improvements to implementation was a theme across 3 studies.^15^^,^^31^^,^^33^ Gidado et al.^31^ suggested that strategies for improved functionality of Xpert machines should be prioritized over the installation of new machines. Secondly, Cattamanchi et al.^15^ reported on the practical benefit of highlighting enabling factors to improve implementation, reporting quality improvement initiatives that positively impacted outcomes along the TB treatment and care cascade including SMS communication for the delivery of Xpert results, and implementing a process to gather monthly performance feedback from health center staff.^15^ Accordingly, sharing of knowledge via standardized reporting provides valuable information that supports the ongoing integration of Xpert for TB testing in LMICs.^13^ In the longer term, consistent reporting and communication of the enabling factors to improve implementation will contribute to systemic improvement in the public health impact of Xpert as programs are scaled up across LMICs.^20^^,^^38^^,^^39^

Sharing of knowledge via standardized reporting provides valuable information that supports the ongoing integration of Xpert for TB testing in LMICs.

### Implementation Science

Implementation frameworks such as the Standards for Reporting Implementation Studies (StARI) and the Consolidated Framework for Implementation Research (CFIR) were developed with the knowledge that effective interventions are often found to be ineffective when implemented in a real-world context.^40^^,^^41^ This is often due to the central role that context plays in understanding how factors such as the social, cultural, economic, political, legal, and physical environment may affect the intervention.^22^ For programs to improve public health impact, there is a need for the continued development of tools and strategies that support successful implementation.^42^ The similar or recurring barriers being experienced across LMIC Xpert interventions indicate the need for more consistent and transparent reporting methods to facilitate knowledge-sharing. Consistent reporting through implementation frameworks will increase understanding of the most effective implementation approaches and contextual influences and enable the scaling up of Xpert interventions.^20^^,^^22^ Therefore, an increase in the use of implementation frameworks for planning and evaluation of Xpert programs has the potential to improve outcomes achieved by Xpert programs and accelerate the translation of research into policy and practice.^42^

### Limitations and Strengths

This review had several limitations. We searched 3 databases and restricted our search to English language publications. Further, of the studies included in this review, only 3 reported being guided by a structured reporting framework^27^^,^^30^^,^^33^ and only 1 was specifically for implementation studies.^33^ Also, not using the word “program” or “programmatic” may have reduced the number of search results. However, this review had several strengths including being guided by PRISMA, as well as the articles for inclusion being appraised by the lead author and 2 co-authors.

## CONCLUSION

With a higher burden of TB in LMICs, implementation research can advance understanding of implementation barriers and enablers. This study demonstrates the commonality of these barriers across geographically dispersed Xpert interventions in LMICs. With greater transparency of these barriers and enablers, program planners can promote a more collaborative approach and adapt interventions to reduce the impact of implementation barriers. To build the evidence base and in turn improve the implementation and effectiveness of Xpert, it is recommended that programs use implementation science frameworks when conducting research and disseminating findings. Wider use of these frameworks will provide valuable insight and support the ongoing improvement of TB programs in LMICs.

## Notes

**Peer Reviewed**

**First published online:** September 8, 2021.
